# Platycodin D alleviates cardiac hypertrophy in the treatment of heart failure by inhibiting p53-dependent BCL-2/Bax/caspase-3 signaling pathway

**DOI:** 10.3389/fphar.2026.1832803

**Published:** 2026-05-26

**Authors:** Yingrui Li, Heng Li, Bin Liu, Yu Li

**Affiliations:** 1 Department of Cardiology, the Second Affiliated Hospital of Chongqing Medical University, Chongqing, China; 2 Department of Intervention, Guiyang Public Health Treatment Center, Guiyang, Guizhou, China

**Keywords:** cardiac hypertrophy, heart failure, network pharmacology analysis, p53, platycodin D

## Abstract

**Introduction:**

Cardiac hypertrophy drives heart failure progression, with cardiomyocyte apoptosis playing a central role. Platycodin D (PLD), a bioactive saponin derived from Platycodon grandiflorum, exhibits cardioprotective properties, but its therapeutic potential and mechanisms in heart failure remain poorly understood.

**Methods:**

In this study, we employed a mouse model of isoprenaline (ISO)-induced heart failure to investigate the therapeutic potential of PLD. Mice were administered PLD at doses of 12.5, 25, or 50 mg/kg/d for 4 weeks to evaluate its effects on cardiac function and hypertrophy. To elucidate the underlying mechanisms, we performed network pharmacology analysis, which identified p53 as a candidate target of PLD. The functional role of p53 was further assessed through pharmacological interventions, comparing the effects of PLD with those of the p53 inhibitor pifithrin-α (PFT-α) and examining whether p53 activation could abrogate PLD-mediated cardioprotection.

**Results:**

PLD dose-dependently improved cardiac function and attenuated cardiac hypertrophy in ISO-induced HF mice. Network pharmacology and molecular docking identified p53 as a direct binding partner of PLD. PLD alleviated the apoptosis of cardiomyocyte by inhibition p53 depended on Bcl-2/Bax/caspase-3 signaling pathway. The cardioprotective effects of PLD were comparable to those of the p53 inhibitor pifithrin-α (PFT-α). But, these benefits were abolished upon p53 activation.

**Conclusion:**

This study demonstrates that PLD attenuates ISO-induced cardiac hypertrophy and heart failure by inhibiting the p53-mediated Bcl-2/Bax/caspase-3 apoptotic pathway. These findings establish PLD as a promising therapeutic candidate for heart failure.

## 1 Introduction

Heart failure (HF) is a significant public health problem worldwide, which brings a heavy burden to the global economy and health system, and also seriously affects the quality of life of patients (Johansson et al., 2021; Ziaeian et al., 2016). Pharmacological interventions, including beta-blockers and angiotensin-converting enzyme inhibitors, remain cornerstone therapies in HF management (Rossignol et al., 2019). The development of novel therapeutics, particularly traditional Chinese medicine, has expanded treatment options for HF patients, with growing evidence supporting their potential to delay disease progression or improve clinical outcomes (Chen et al., 2023).

Platycodon grandiflorus, is a perennial herb from the Platycodon genus (Ma et al., 2024). Platycodin D (PLD), the primary bioactive saponin derived from Platycodon grandiflorus, exhibits diverse pharmacological properties, including antioxidant, anti-apoptotic, and anti-inflammatory effects, underscoring its potential therapeutic role in cardiovascular diseases (Xie et al., 2023). In the hypoxia/reoxygenation model of cardiomyocytes, PLD activated the Akt/Nrf2/HO-1 signaling pathway, mitigating oxidative stress and apoptosis (Wang et al., 2018). In type 2 diabetic mice, PLD effectively ameliorated early atherosclerotic inflammation and lipid translocation deposition (Wang et al., 2025). And PLD also increased NO production and later inhibited endothelial adhesion to monocytes (Wu et al., 2012). Cardiac hypertrophy is initially an adaptive response of the heart to increased workload, characterized by increased cardiomyocyte size and ventricular wall thickening (Nakamura et al., 2018). However, prolonged pathological stimulation can lead to a state of decompensation, leading to heart failure (Tham et al., 2015; Li et al., 2022). Oxidative stress, inflammation, and apoptosis are key pathological processes that drive cardiac hypertrophy, thereby accelerating the progression to heart failure (Li et al., 2022; Higashikuni et al., 2023; Ji et al., 2022). Combined with the multiple effects of PLD, we hypothesized that PLD treatment could alleviate cardiac hypertrophy and mitigate heart failure.

TP53, a tumour suppressor gene extensively studied in cardiovascular disease, exerts its function through its encoded protein, p53 (Huang et al., 2024). As a critical transcription factor, p53 plays an indispensable role in preserving genomic integrity by initiating DNA repair or inducing apoptosis in response to DNA damage, thereby preventing the propagation of potentially harmful mutations (Zhang et al., 2024). Dysregulated TP53 signaling contributes to cardiac hypertrophy through diverse mechanisms. In diabetic cardiomyopathy mice, TP53 inhibition alleviated hypertrophy and inflammation by suppressing TNF and TGF-β1 pathways (Fang et al., 2024). Furthermore, in pressure-overload models, TP53 accumulation was shown to exacerbate hypertrophy by inhibiting Hif-1-dependent angiogenesis, ultimately leading to cardiac deterioration (Sano et al., 2007). The increased TP53 also activated the Bcl-2/Bax/caspase-3 apoptosis signaling pathway in cardiomyocytes (Wang et al., 2019). In contrast, inhibiting TP53 blocked this pathway, reduced cardiomyocyte apoptosis, and improved cardiac hypertrophy (Huang et al., 2025). In H2O2-induced premature senescence of fibroblast cells, PLD exerted protective functions by inhibiting p53 (Shi et al., 2020). In this study, we elucidated the molecular mechanism underlying the therapeutic effects of PLD on isoprenaline-induced heart failure by integrating network pharmacology with experimental validation. And we demonstrated that PLD exerted its anti-hypertrophic effects primarily by modulating p53.

## 2 Materials and methods

### 2.1 Animal models

**FIGURE 1:**
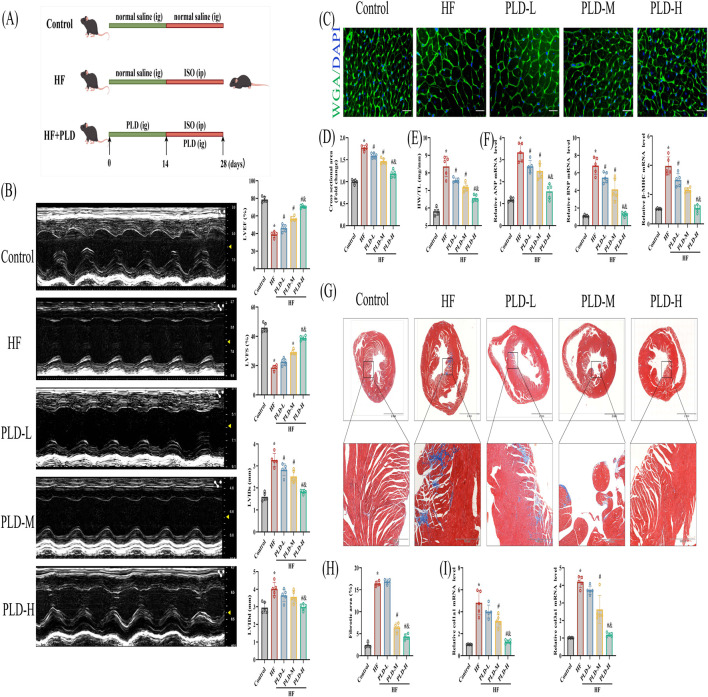
Platycodin D (PLD) ameliorates isoprenaline (ISO)-induced heart failure in mice. **(A)** Schematic of the experimental protocol. **(B)** Representative cardiac ultrasound images and quantitation results are presented for mice subjected to 2 weeks of PLD pretreatment (at varying doses) followed by 2 weeks of combined ISO co-intervention. Measured parameters include left ventricular ejection fraction (LVEF), left ventricular fractional shortening (LVFS), left ventricular internal diameter at systole (LVIDs), and left ventricular internal diameter at diastole (LVIDd). **(C)** Representative wheat germ agglutinin (WGA) staining images of cardiac tissue sections reveals group-specific differences in cardiomyocyte hypertrophy. Scale bar = 20 µm. **(D)** Quantification of cardiomyocyte cross-sectional area of different groups (n = 5). **(E)** Heart weight-to-tibial length (HW/TL) ratios were calculated to assess cardiac hypertrophy across experimental groups (n = 5). **(F)** The mRNA expression levels of atrial natriuretic peptide (ANP), brain natriuretic peptide (BNP), and β-myosin heavy chain (β-MHC) in mice cardiac tissues across experimental groups (n = 5). **(G)** Representative Masson’s trichrome staining images of cardiac tissue sections in different groups. Blue: collagen; red: cardiomyocytes. Scale bar = 2 mm. **(H)** The fibrotic area in cardiac tissue sections was quantified from Masson’s trichrome-stained images (n = 4). **(I)** The mRNA expression levels of collagen type I alpha 1 (col1a1) and collagen type III alpha 1 (col3a1) in mice cardiac tissues of different groups (n = 5). *P < 0.05 vs. Control, #P < 0.05 vs. HF, & P < 0.05 vs. HF + PLD-M.

Animal experiments were carried out with the approval of the Ethics Committee of the Second Affiliated Hospital of Chongqing Medical University (Approval No: IACUC-SAHCQMU-2025-0249). C57/BL 6J mice were provided by the Laboratory Animal Centre of Chongqing Medical University (Chongqing, China). Eight-week-old C57BL/6J mice of either sex were raised in the animal centre at a temperature of approximately 22 °C and had free access to food and tap water. The method of inducing heart failure with ISO was the same as before (Li et al., 2023). Briefly, mice received an intraperitoneal injection of ISO (7.5 mg/kg/d) for 14 days. PLD (MCE, China) was dissolved and diluted in normal saline. In the treatment groups, mice received separate intragastric administrations of low (12.5 mg/kg/d, PLD-L), medium (25 mg/kg/d, PLD-M), and high (50 mg/kg/d, PLD-H) doses of PLD for 14 days, followed by another 14 days of co-administration with ISO. Equal volumes of normal saline were used as a vehicle control. The experimental design was illustrated in Figure 1A. For further experiments, pifithrin-α (PFT-α, a p53 inhibitor, 5 mg/kg/day, MCE, China) or NSC697923 (NSC, a p53 agonist, 2 mg/kg/day, MCE, China) was intraperitoneally administered to mice for two consecutive weeks, starting at the onset of modelling (Zhang et al., 2025). And PFT-α was dissolved using dimethyl sulfoxide (DMSO) as the solvent.

### 2.2 Assessment of cardiac function

To assess heart function, a cardiac echocardiogram was performed using Vevo 3100LT (VisualSonics, Fujifilm, Tokyo, Japan). The mice were anesthetized via inhalation of 2% isoflurane in an induction chamber and subsequently positioned in a supine posture on the operating table. Anesthesia was maintained through continuous inhalation via a face mask, with the anesthetic concentration adjusted to sustain a heart rate of 400-500 beats per minute. High-quality, representative M-mode echocardiographic images were acquired. Left ventricular end-diastolic diameter (LVIDd) and end-systolic diameter (LVIDs) were measured using VevoLab software, and the ejection fraction (EF%) and left ventricular fractional shortening (FS%) were derived from these measurements. All procedures were performed by a single operator who was blinded to the experimental group assignments of the mice.

### 2.3 Masson trichrome staining

For Masson Trichrome Staining, the hearts were harvested after perfusion with cold saline and fixed in 4% paraformaldehyde solution. Following dehydration, embedding, and sectioning, paraffin-embedded heart sections were prepared. The sections were then subjected to dewaxing and antigen retrieval. Masson trichrome staining was performed according to the manufacturer’s protocol (Solaibao, Beijing, China), resulting in the fibrotic areas being distinctly stained blue. The percentage of fibrosis area in the stained heart sections was quantified using ImageJ software.

### 2.4 Wheat germ agglutinin (WGA) staining

The cross-sectional area of cardiomyocytes was assessed by labeling the cell membrane with WGA staining. Following antigen repair, the sections were incubated with WGA working solution (Invitrogen, United States) in the dark for 1 h at 37 °C. Then, the sections were rinsed three times in PBS solution for 5 min each time, and subsequently incubated with DAPI solution (Invitrogen, United States) for 2 min to facilitate nuclear staining. The stained sections were visualized under a fluorescence microscope to capture high-quality representative images. Quantitative analysis of the images was performed using ImageJ software.

### 2.5 Potential targets prediction for platycodin D

The chemical structure of PLD (in SMILES format) was obtained from the PubChem database and then imported into the SwissTargetPrediction (http://.swisstargetprediction.ch/) and SEA (http://sea.docking.org/) databases to predict potential drug targets. We also imported PLD into the ChEMBL (https://pubchem.ncbi.nlm.nih.gov/source/ChEMBL) database to predict potential drug targets. ChEMBL targets with reported activity (IC_50_ ≤ 10 uM) were considered for further analysis. When determining the retrieval targets, we limited the species to *Homo sapiens*, and required a prediction probability >0.1. And a search was performed in the GeneCards databases using the keywords “cardiac hypertrophy” and “myocardial hypertrophy”. Disease targets were selected based on a relevance score ≥ the median value. Subsequently, disease targets were merged, and any duplicates were eliminated. The common targets were identified by intersecting drug targets and disease targets using the WeiShengXin platform (https://www.bioinformatics.com.cn/), which revealed potential targets for PLD treatment of cardiac hypertrophy.

### 2.6 The building of protein-protein interaction (PPI) network

Common targets were searched in the STRING database (https://string-db.org), and a PPI network was established with a minimum interaction score threshold of 0.4. Topological analysis was performed using Cytoscape 3.10.0 software, and targets whose degree values were at least twice the median were selected as core targets.

### 2.7 GO and KEGG enrichment analysis

The common targets of diseases and drugs were imported into the WeiShengXin platform (https://www.bioinformatics.com.cn/). The top ten signaling pathways identified in GO and KEGG analyses were separately visualized using bioinformatics. GO included biological processes, cellular components, and molecular functions.

### 2.8 Molecular docking verification and dynamics simulation

The top six core intersection targets of diseases and drugs were imported into the CB-DOCK2 (https://cadd.labshare.cn/cb-dock2/php/index.php) databases to verify the possibility of their combination with PLD. It was generally believed that when the binding energy was lower than -5 kJ/mol, it indicated that the ligand had good binding ability with the receptor.

GROMACS 2022 was used for molecular dynamics simulations, and the force field parameters were generated using the pdb2gmx tool of GROMACS. In this study, the Sobtop software was used to generate the topology file for the GAFF2 force field based on the ligand structure. The charge distribution of the ligand was then carried out using the RESP method, ensuring that the charge distribution conforms to the ligand’s physicochemical characteristics. The receptor protein was parameterized using the AMBER14SB force field. The transferable intermolecular potential 3P (TIP3P) water model was employed for system solvation, and a cubic water box was used for solvation, collectively to ensure sufficient solvation and electrical neutrality of the system. To ensure electrical neutrality of the system, 0.15 M NaCl was added to the simulation box via the ‘gmx genion’ script. The long-range electrostatic interactions were calculated using the particle mesh ewald (PME) method with a cutoff distance of 1 nm. Bond lengths are constrained using the LINCS algorithm. Before the molecular dynamics simulation, the system underwent an energy optimization process. Using the Nosé–Hoover coupling, the temperature for the simulation was maintained at 310 K. Using the Parrinello–Rahman pressure coupling, the pressure was maintained at 1 bar. The simulation time was set to 100 ns. The simulation of the system was performed under constant NPT conditions, which were both isothermal and isobaric, with a time step of 2 fs. Finally, root-mean-square deviation (RMSD), root-mean-square fluctuation (RMSF), hydrogen bonds (HBonds), radius of gyration (Rg) and solvent accessible surface area (SASA) were calculated using GROMACS correlation tools, and the stability, structural changes and solvent effects of the system were analyzed.

### 2.9 Western blotting analysis

Proteins were extracted from heart tissues using RIPA lysis buffer, and the protein concentration was later measured using a BCA kit (Sulaibao, Beijing, China). Proteins from heart tissues were separated using SDS-PAGE and subsequently transferred to PVDF membranes. These membranes were incubated with an antibody blocking solution (Sulaibao, Beijing, China) for 30 min at room temperature, then incubated with the primary antibody overnight, and subsequently incubated with the secondary antibody solution for 1 h at room temperature the following day. The primary antibodies included p53, BCL-2, Bax, and caspase-3 (CST, Shanghai, China) and were diluted at 1:1000. Results were acquired using the Odyssey infrared imaging system (Li-Cor Biosciences, Lincoln, NE, United States) and analyzed with ImageJ software.

### 2.10 Quantitative real-time PCR analysis

Total RNA extraction from mouse heart tissues was performed using TRIzol from Invitrogen, United States. The relative levels of mRNA expression for each gene were quantified using SYBR Green detection. To ensure accuracy, duplicate wells were set for each sample, and the final results were averaged. The 2^−ΔΔCT^ method was employed to quantify the relative expression of each gene, with GAPDH serving as the reference for normalization. The sequences of primers for each gene are listed in Supplementary Table S1.

### 2.11 Data analysis

Statistical analyses were performed using SPSS software, with results presented as mean ± standard error of the mean (SEM). The choice between parametric and non-parametric tests was determined based on the outcomes of normality testing. For comparisons involving more than two groups, a one-way analysis of variance (ANOVA) was conducted, followed by the Holm-Sidak *post hoc* test. A P-value of less than 0.05 was established as the threshold for statistical significance.

## 3 Results

### 3.1 Platycodin D significantly improved the cardiac function in mice with ISO-induced heart failure

We established a mouse model of HF using a previously established method. Specifically, mice received an intraperitoneal injection of isoprenaline at a dose of 7.5 mg/kg daily for 2 weeks. After ISO administration, the mice exhibited an apparent HF phenotype, characterized by decreased EF and FS, and increased LVIDs and LVIDd, as determined by cardiac ultrasound examination (Figure 1B). At the same time, mice also exhibited significant cardiac hypertrophy, as evidenced by WGA staining (Figure 1C), which revealed an increase in cardiomyocyte cross-sectional area (Figure 1D), a rise in the HW/TL ratio (Figure 1E), and an increase in the expression of cardiac hypertrophy markers (ANP, BNP, and β-MHC) (Figure 1F). To investigate the therapeutic potential of PLD in HF, mice were pretreated with PLD via oral gavage for 2 weeks, followed by co-administration with ISO for an additional 2 weeks (Figure 1A). Our results demonstrated that in HF mice, increasing doses of PLD led to a significant elevation in EF and FS, accompanied by a reduction in LVIDs and LVIDd (Figure 1B). Furthermore, PLD treatment dose-dependently decreased the cross-sectional area of cardiomyocytes HW/TL ratio, and downregulated the expression of cardiac hypertrophy markers (Figures 1C–F). Additionally, Masson trichrome staining revealed that PLD administration dose-dependently attenuated the area of cardiac fibrosis in the treated groups (Figures 1G–I). Therefore, we aim to investigate the mechanism by which PLD attenuates cardiac hypertrophy and thereby improves HF.

### 3.2 Network pharmacology analysis results

**FIGURE 2:**
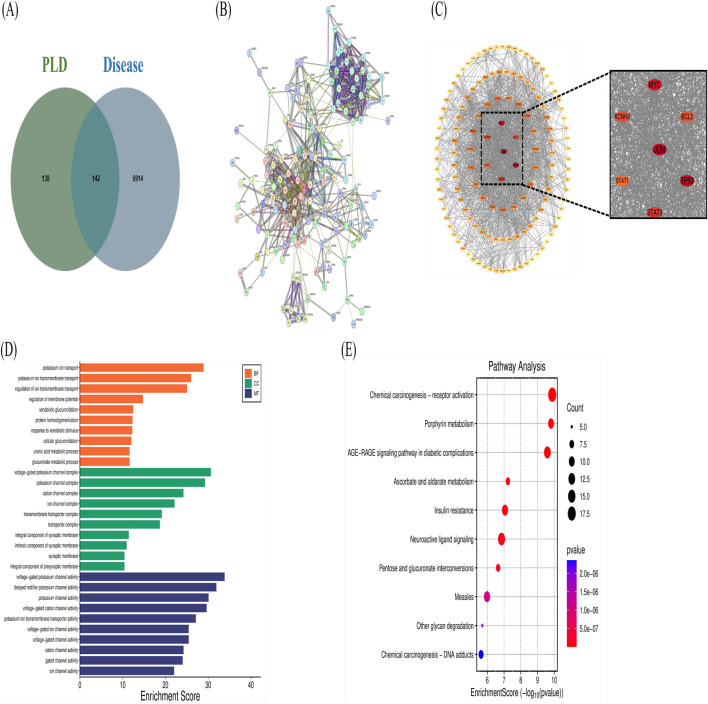
Results of network pharmacology analysis. **(A)** Venn diagram showing the overlapping targets of PLD and cardiac hypertrophy. **(B)** Protein-protein interaction (PPI) network of the shared targets between PLD and cardiac hypertrophy. **(C)** PPI network visualized using Cytoscape, where node color intensity correlates with degree centrality (darker nodes indicate higher connectivity). **(D)** Top 10 significantly enriched Gene Ontology (GO) terms. **(E)** Top 10 significantly enriched Kyoto Encyclopedia of Genes and Genomes (KEGG) pathways.

We conducted network pharmacology analysis to identify the genes targeted by PLD (Lee et al., 2025). A total of 280 PLD target genes were identified through database searches (Figure 2A; Supplementary Table S2). Then, we searched for disease target genes using the terms “cardiac hypertrophy” and “myocardial hypertrophy”, respectively (Supplementary Tables S3-S4). After combining and removing duplicate genes, we obtained 7056 disease targets (Figure 2A; Supplementary Table S5). Among these genes, 142 genes overlapped with PLD target genes (Figure 2A; Supplementary Table S6). We constructed PPI networks using the STRING database and visualized the results using Cytoscape software (Figures 2B,C). The core targets were identified by ranking the degree of each target in PPI networks; therefore, these targets, including JUN, TP53, MYC, STAT3, BCL2, and KCNH2, were recognized as core targets.

### 3.3 GO and KEGG pathway enrichment analysis

GO analysis results showed that there were 1,355 biological processes (BP) entries, mainly involving potassium ion transport, regulation of ion transmembrane transport and regulation of membrane potential (p < 0.05, Supplementary Table S7). 101 cell composition (CC) entries mainly involving voltage-gated potassium channel complex, potassium channel complex, and cation channel complex (p < 0.05, Supplementary Table S8). And 116 molecular function (MF) entries mainly involving voltage-gated potassium channel activity, delayed rectifier potassium channel activity, and potassium channel activity (p < 0.05, Supplementary Table S9). In Figure 2D, the top 10 BP, CC, and MF entries were arranged in ascending order of P-value and displayed as a visual bubble plot (Figure 2D). A total of 131 pathways were identified following KEGG enrichment analysis (p < 0.05, Supplementary Table S10), and the top 10 were considered key pathways, for which visual analysis was performed (Figure 2E). And the core genes were mainly enriched in chemical carcinogenesis-receptor activation, porphyrin metabolism, and the AGE-RAGE signaling pathway. Notably, most of these pathways were associated with core genes, including JUN, TP53, MYC, STAT3, and BCL2.

### 3.4 Molecular docking and dynamics simulation results

**FIGURE 3:**
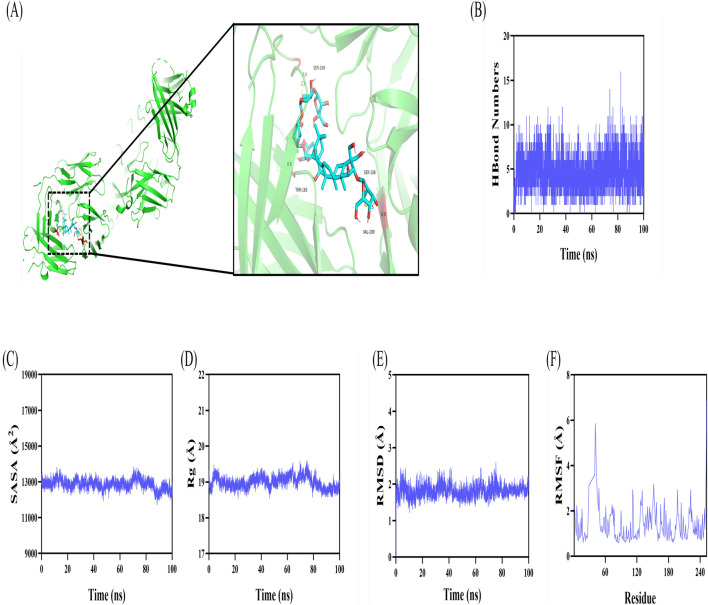
Molecular docking and dynamics simulation analysis of the PLD-p53 interaction. **(A)** Molecular docking results showing the high-affinity binding pose of PLD with p53. **(B)** Time-dependent evolution of hydrogen bond interactions between PLD and p53 during the molecular dynamics simulation. **(C)** Solvent accessible surface area (SASA) of the PLD-p53 complex as a function of simulation time. **(D)** Radius of gyration (Rg) of the PLD-p53 complex over the simulation trajectory. **(E)** Root-mean-square deviation (RMSD) of the PLD-p53 complex backbone atoms relative to the initial structure. **(F)** Root-mean-square fluctuation (RMSF) of PLD-p53 complex residues, reflecting local flexibility.

Molecular docking of PLD was performed with the proteins encoded by JUN, TP53, MYC, STAT3, and BCL2, respectively. The results showed that PLD exhibited the lowest binding energy with p53 (encoded by TP53) among the five core proteins (Supplementary Table S11), suggesting a strong binding affinity between PLD and p53. Moreover, PLD is predicted to bind to p53, interacting with specific residues (Val-109, Thr-165, Ser-168, Ser-108) via hydrogen bonds (Figure 3A). The molecular dynamics simulation results demonstrated that the PLD-p53 complex exhibited stability throughout the simulation period, as evidenced by consistent hydrogen bonding, favourable solvent accessibility, and minimal RMSD fluctuations (Figures 3B–E). Additionally, the RMSF values of the complex were relatively low, suggesting reduced flexibility and enhanced structural stability (Figure 3F). Based on these findings, we identified p53 as the primary target of PLD in ameliorating cardiac hypertrophy and proceeded with experimental validation.

### 3.5 Pharmacological inhibition of p53 attenuates cardiac hypertrophy in mice with ISO-induced heart failure

**FIGURE 4:**
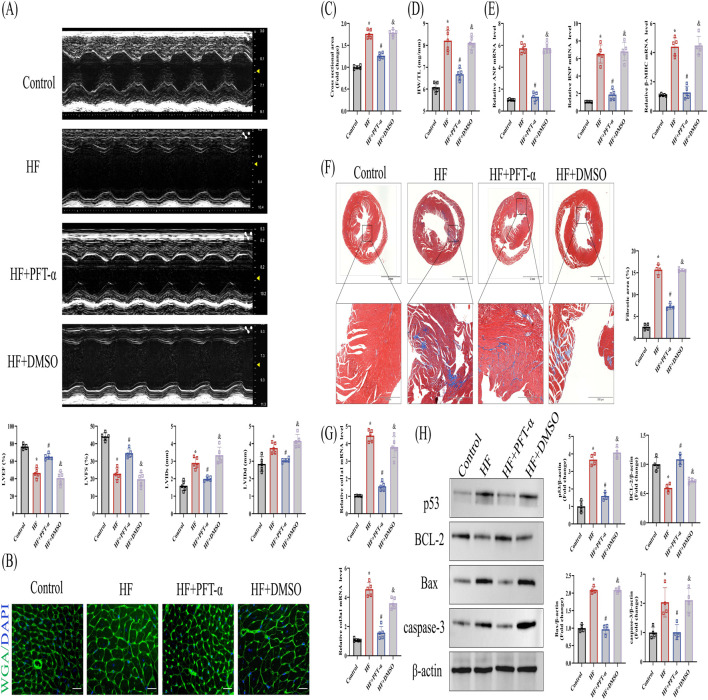
Pharmacological inhibition of p53 by PFT-α alleviates cardiac hypertrophy by modulating apoptotic pathways. **(A)** Inhibition of p53 improves cardiac function, as demonstrated by increased LVEF, LVFS, and decreased LVIDs and LVIDd (n = 5). **(B)** Representative WGA-stained cardiac sections. Scale bar = 20 µm. **(C)** Inhibition of p53 reverses ISO-induced increase of cardiomyocyte cross-sectional area (n = 5). **(D)** Inhibition of p53 reduces the HW/TL ratio (n = 5). **(E)** Inhibition of p53 suppresses hypertrophic markers, downregulating ISO-induced mRNA expression of ANP, BNP, and β-MHC in cardiac tissues (n = 5). **(F)** Inhibition of p53 attenuates cardiac fibrosis, as quantified by fibrotic area (n = 4). **(G)** Inhibition of p53 downregulates fibrotic markers, reducing ISO-induced increases in col1a1 and col3a1 at the mRNA level in cardiac tissues (n = 5). **(H)** Inhibition of p53 reduces the levels of pro-apoptotic proteins (Bax and caspase-3) while increasing the expression of the anti-apoptotic protein Bcl-2 in cardiac tissues (n = 4). *P < 0.05 vs. Control, #P < 0.05 vs. HF, & P < 0.05 vs. HF + PET-α.

From the results of network pharmacology, molecular docking, and dynamics simulation analysis, we found that p53 played an essential role in the process of cardiac hypertrophy. Therefore, we utilized the PFT-α, an inhibitor of p53, to investigate the functional role of p53 in cardiac hypertrophy and its underlying mechanism. In the model group, ISO treatment significantly upregulated p53 expression in myocardial tissues (Figure 4H). Conversely, PFT-α treatment effectively suppressed this ISO-induced upregulation of p53 in myocardial tissues (Figure 4H). This result suggested a role for p53 in ISO-induced cardiac hypertrophy. The inhibition of p53 improved the cardiac function in mice with ISO-induced HF, as exhibited by increased LVEF and LVFS, and decreased LVIDs and LVIDd (Figure 4A). Subsequently, we observed the effect of p53 inhibition on cardiac hypertrophy in HF mice. As illustrated in Figures 4B–E, PFT-α treatment significantly attenuated cardiac hypertrophy, as evidenced by reduced cardiomyocyte cross-sectional area, decreased HW/TL ratio, and downregulated mRNA expression of hypertrophic markers (ANP, BNP, and β-MHC) in cardiac tissues of mice with heart failure. We also examined the effect of p53 inhibition on cardiac fibrosis. The fibrotic area of the heart in the PFT-α treatment group was lower than in the HF group (Figure 4F), and the expression of fibrosis markers (Col1a1 and Col3a1) was also lower (Figure 4G). Apoptosis plays a critical role in the pathological progression of cardiac hypertrophy and serves as a central mechanism underlying its transition to HF (Foo et al., 2006). In the apoptotic process, p53 serves as a pivotal regulator—its activation promotes the upregulation of pro-apoptotic molecules and suppresses anti-apoptotic factors. As illustrated in Figure 4H, sustained ISO treatment markedly increased the expression of the pro-apoptotic proteins Bax and caspase-3 in cardiac tissues, while concurrently reducing levels of the anti-apoptotic protein BCL-2. Significantly, PFT-α effectively counteracted these ISO-induced changes in protein expression. These results indicated that p53 downregulation in the cardiac tissues of ISO-induced HF mice attenuated apoptosis, alleviated cardiac hypertrophy, and ultimately improved cardiac function.

### 3.6 PLD exerts its anti-hypertrophic effects by inhibiting the p53-regulated BCL-2/Bax/caspase-3 apoptotic signaling pathway

**FIGURE 5:**
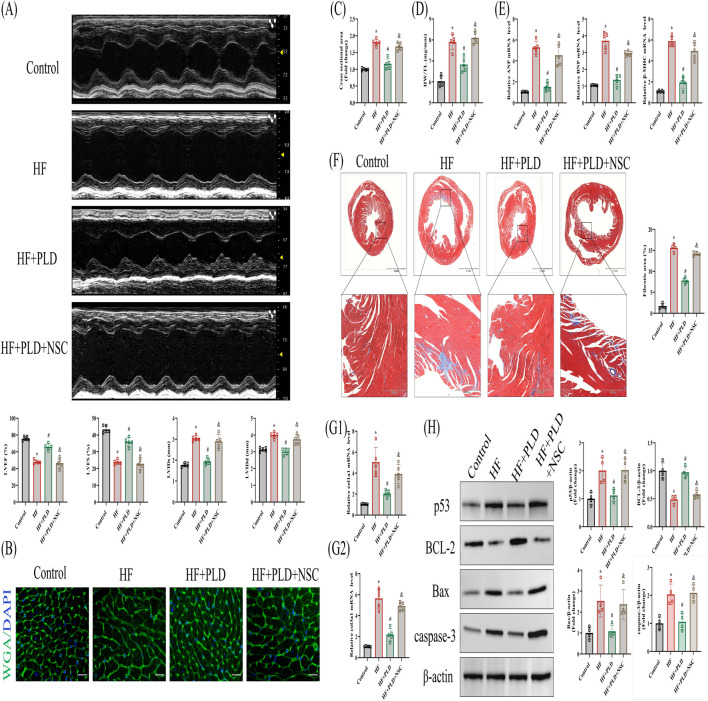
PLD attenuates ISO-induced cardiachypertrophy by blocking the p53-driven BCL-2/Bax/caspase-3 apoptotic signaling axis. **(A)** NSC697923 (NSC)-mediated activation of p53 abrogated the cardioprotective effects of PLD, as verified by decreased LVEF, LVFS, and increased LVIDs and LVIDd (n = 5). **(B)** Representative cardiac sections stained with WGA. Scale bar = 20 µm. **(C)** Activation of p53 counteracted the inhibitory effect of PLD on cardiomyocyte hypertrophy, as demonstrated by an increase in cardiomyocyte cross-sectional area (n = 5). **(D)** Activation of p53 results in a significant elevation of the HW/TL ratio (n = 5). **(E)** Activation of p53 increases the expression of cardiac hypertrophic markers (ANP, BNP, and β-MHC) in cardiac tissues (n = 5). **(F)** Activation of p53 exacerbated cardiac fibrosis, as indicated by an increase in the fibrotic area (n = 4). **(G)** Activation of p53 upregulates the expression of fibrosis markers (col1a1 and col3a1) in cardiac tissues (n = 5). **(H)** Activation of p53 promoted apoptosis by upregulating pro-apoptotic proteins (Bax and caspase-3) while downregulating the anti-apoptotic protein Bcl-2 in cardiac tissues (n = 4). *P < 0.05 vs. Control, #P < 0.05 vs. HF, & P < 0.05 vs. HF + PLD.

Our findings demonstrated that p53 served as a crucial target of PLD in cardiac hypertrophy. Furthermore, pharmacological inhibition of p53 has been shown to suppress apoptosis, ameliorate cardiac hypertrophy, and improve cardiac function in ISO-induced HF mice. To clarify the role of p53 in PLD-mediated cardioprotection, we utilized the p53 agonist NSC to investigate whether p53 activation affects the efficacy of PLD in reducing cardiac hypertrophy and improving cardiac function. In HF mice, PLD treatment reduced p53 levels in cardiac tissue; however, co-treatment with NSC restored p53 expression (Figure 5H). This result suggested a regulatory role for p53 in the mechanism of action of PLD. The activation of p53 by NSC counteracted the beneficial effect of PLD on cardiac function, as evidenced by decreased LVEF and LVFS, and increased LVIDs and LVIDd (Figure 5A). PLD exerts a protective effect against cardiac hypertrophy; however, co-treatment with NSC abrogates these benefits, as indicated by significant increases in cardiomyocyte area (Figures 5B,C), HW/TL ratio (Figure 5D), and the expression of hypertrophic markers in heart tissues (ANP, BNP, and β-MHC) (Figure 5E). Similarly, the administration of NSC also counteracted the ameliorative effect of PLD on cardiac fibrosis. Quantitative analysis revealed a significant increase in the area of cardiac fibrosis and elevated expression of fibrotic markers (col1a1 and col3a1) in the NSC-treated group compared to the PLD-treated group (Figures 5F,G). The BCL-2/Bax/caspase-3 signaling pathway is a p53-mediated apoptotic signaling pathway, which is closely related to cardiac hypertrophy (Roy et al., 2005). Western blot analysis demonstrated that NSC counteracts PLD-mediated inhibition of the apoptotic signaling pathway by upregulating pro-apoptotic proteins (Bax and caspase-3) and downregulating anti-apoptotic proteins (Bcl-2) (Figure 5H). Taken together, the anti-hypertrophic action of PLD is mediated through inhibition of the p53-regulated BCL-2/Bax/caspase-3 apoptosis signaling pathway. Furthermore, the activation of p53 can effectively reverse this cardio-protective effect of PLD.

## 4 Discussion

Despite advances in treatment, heart failure (HF) continues to pose a major public health challenge, driving the search for novel therapeutic agents such as those derived from traditional Chinese medicine (Willerson et al., 2019; Jia et al., 2020; Hao et al., 2017). PLD, a bioactive natural product with well-documented anti-apoptotic and anti-oxidative properties, has shown promise in various disease models, including cardiovascular disease (Chen et al., 2022; Suh et al., 2018; Zhang et al., 2023). Here, we extend these findings by demonstrating that PLD attenuates cardiac dysfunction in a mouse model of isoproterenol (ISO)-induced HF. This cardioprotection was achieved primarily through the suppression of pathological cardiac hypertrophy. Mechanistically, PLD treatment inhibited p53 activity, resulting in decreased expression of pro-apoptotic proteins (Bax and cleaved caspase-3) and increased expression of the anti-apoptotic protein Bcl-2.

Cardiac remodeling, a pathological foundation for heart failure (HF) progression, involves complex interactions among multiple cell types, including cardiomyocytes, fibroblasts, and immune cells. Among these, cardiomyocytes serve as the primary determinants of cardiac function, making them a critical therapeutic target for the treatment of HF (Meng et al., 2024). In the present study, we established a mouse model of HF via sustained isoprenaline (ISO) stimulation, which reliably induces β-adrenergic receptor-mediated cardiac dysfunction (Fu et al., 2025). This was evidenced by significant reductions in EF and FS, along with increased LVIDs and LVIDd. Consistent with previous reports (Peng et al., 2024), these functional impairments were accompanied by marked cardiac hypertrophy, as confirmed by increased HW/TL ratio, enlarged cardiomyocyte cross-sectional area, and upregulation of hypertrophic marker genes. Since persistent hypertrophy precedes and exacerbates HF (Croon et al., 2022), targeting hypertrophy represents a viable therapeutic strategy for heart failure.

PLD has demonstrated cardioprotective properties; however, its therapeutic potential in heart failure and cardiac hypertrophy remains largely unexplored. In the present study, we show that PLD treatment dose-dependently attenuates cardiac hypertrophy and fibrosis, leading to improved cardiac function in a mouse model of (ISO-induced heart failure. To identify the molecular targets mediating these effects, we employed a network pharmacology approach, which yielded 142 genes commonly associated with both PLD and cardiac hypertrophy. Subsequent molecular docking and molecular dynamics simulations identified TP53 as a high-confidence binding partner, demonstrating stable complex formation through multiple hydrogen bonds. These findings establish TP53 as a key functional target of PLD in the context of cardiac hypertrophy, providing a mechanistic basis for its therapeutic effects in HF.

The transcription factor p53 (encoded by TP53) is a central mediator of cardiovascular disease, governing critical processes such as apoptosis, oxidative stress, and ferroptosis. In the model of pressure overload-induced cardiac hypertrophy, p53 was consistently upregulated, and its genetic deletion attenuated pathological remodeling (Li et al., 2018). Network pharmacology and molecular docking analyses identified p53 as a direct binding partner of PLD. Consistent with the established role of p53 in cardiac remodeling, we observed significant p53 activation in ISO-induced heart failure mice. Pharmacological inhibition of p53 using PFT-α effectively alleviated cardiac hypertrophy and improved cardiac function. Critically, co-administration of a p53 agonist completely abolished PLD’s protective effects on cardiac hypertrophy, fibrosis, and function, and reversed PLD-induced suppression of p53 expression. Together, these findings indicated that PLD ameliorated ISO-induced cardiac hypertrophy and heart failure primarily through targeted inhibition of the p53 pathway.

Cardiomyocyte apoptosis is a key pathological driver in the progression from cardiac hypertrophy to heart failure (Jin et al., 2023; Taniike et al., 2008). Although cardiac hypertrophy initially serves as an adaptive response to stress, its persistence leads to maladaptive remodeling and eventual cardiac dysfunction. The p53 serves as a central regulator of cardiomyocyte apoptosis, operating through multiple downstream pathways (Toko et al., 2010; Liu et al., 2024). Among these, the p53-Bcl-2/Bax axis is particularly critical in apoptosis regulation (Bai et al., 2020). Mechanistically, p53 activation promotes apoptosis by upregulating the pro-apoptotic protein Bax while suppressing the anti-apoptotic protein Bcl-2 (Chipuk et al., 2004). This shift in the Bax/Bcl-2 ratio subsequently activates caspase-3, a key executor of apoptosis, thereby triggering programmed cell death (Yang et al., 2002; Yang et al., 2001). In line with this established mechanism, our study demonstrated that PLD treatment significantly decreased p53 expression in the hypertrophic myocardium of heart failure mice, accompanied by downregulation of pro-apoptotic proteins (Bax and caspase-3) and upregulation of the anti-apoptotic protein Bcl-2. Notably, co-administration of a p53 agonist mostly abolished these regulatory effects, confirming the p53-dependence of PLD’s anti-apoptotic action. Collectively, these findings demonstrate that PLD attenuated cardiac hypertrophy and improved cardiac function in HF mice by modulating the Bcl-2/Bax/caspase-3 apoptotic pathway through p53-dependent mechanisms.

This study provides evidence for the therapeutic efficacy of PLD in ISO-induced heart failure through suppression of cardiac hypertrophy and p53-mediated apoptotic signaling. However, several limitations merit consideration. The use of a single HF model limits the generalizability of our findings, underscoring the need for future investigations in diverse HF models to establish the broader applicability of PLD. Additionally, while network pharmacology analysis served as a powerful tool for uncovering drug mechanisms, its reliability depends on the completeness and accuracy of the underlying databases. Potential biases or gaps in these databases may introduce uncertainties into the findings. Future research should also prioritize confirming the safety and efficacy of PLD in preclinical and clinical settings to advance its potential application in disease treatment. Addressing these limitations in future work will be critical for advancing PLD toward clinical application in HF management.

## 5 Conclusion

In conclusion, this study demonstrates that PLD effectively ameliorates ISO-induced heart failure by attenuating cardiac hypertrophy. Mechanistically, PLD exerts its cardioprotective effects through suppression of the p53-mediated Bcl-2/Bax/caspase-3 apoptotic pathway. These findings highlight PLD as a promising therapeutic candidate for heart failure.

## Data Availability

The data presented in this study were derived from the PubChem database (https://pubchem.ncbi.nlm.nih.gov/taxonomy/94286), the GeneCards database (using keywords "cardiac hypertrophy" and "myocardial hypertrophy"), the STRING database (https://string-db.org) for PPI analysis, the WeiShengXin platform (https://www.bioinformatics.com.cn/) for GO and KEGG analyses, and the CB-DOCK2 database (https://cadd.labshare.cn/cb-dock2/php/index.php) for molecular binding validation with PLD. Detailed methods are described in the Methods section.
